# Is coronary multivessel disease in acute myocardial infarction patients still associated with worse clinical outcomes at 1‐year?

**DOI:** 10.1002/clc.23567

**Published:** 2021-02-14

**Authors:** Etienne Puymirat, Ariel Nakache, Christophe Saint Etienne, Pierre Marcollet, Olivier Fichaux, Marie‐Pascale Decomis, Stephan Chassaing, Philippe Commeau, Nicolas Danchin, Guillaume Cayla, Gilles Montalescot, Hakim Benamer, Rene Koning, Pascal Motreff, Grégoire Rangé

**Affiliations:** ^1^ Department of Cardiology Assistance Publique‐Hôpitaux de Paris (AP‐HP), Hôpital Européen Georges Pompidou Paris France; ^2^ Université de Paris Paris France; ^3^ Cardiology Department Centre Hospitalo‐Universitaire de Tours Tours France; ^4^ Cardiology Department Centre Hospitalier de Bourges Bourges France; ^5^ Cardiology Department Centre Hospitalo‐régional d'Orléans Orléans France; ^6^ Cardiology Department Clinique Oréliance Saran France; ^7^ Cardiology Department Nouvelle Clinique Tourengelle Tours France; ^8^ Cardiology Department Polyclinique les Fleurs Ollioules France; ^9^ Cardiology Department CHU Nîmes, Université Montpellier Nîmes France; ^10^ Cardiology Department Groupe hospitalier Pitié‐Salpêtrière Paris France; ^11^ Cardiology Department Clinique de la Roseraie; ICPS Massy Ramsay group Paris 13 France; ^12^ Cardiology Department Clinique Saint‐Hilaire Rouen France; ^13^ Cardiology Department Centre Hospitalo‐Universitaire de Clermont‐Ferrand Clermont Ferrand France; ^14^ Cardiology Department Les Hôpitaux de Chartres Chartres France

**Keywords:** acute myocardial infarction, multivessel disease, primary percutaneous coronary intervention, ST‐elevation myocardial infarction

## Abstract

**Background:**

ST‐elevation myocardial infarction (STEMI) patients with multivessel disease (MVD) are associated with a worse prognosis. However, few comparisons are available according to coronary status in the era of modern reperfusion and optimized secondary prevention.

**Hypothesis:**

We hypothesized that the difference in prognosis according to number of vessel disease in STEMI patients has reduced.

**Methods:**

All consecutive STEMI patients undergoing primary percutaneous coronary intervention (PCI) within 24 h of symptoms onset between January 1, 2014 and June 30, 2016 enrolled in the CRAC (Club Régional des Angioplasticiens de la région Centre) France PCI registry were analyzed. Baseline characteristics, management, and outcomes at 1‐year were analyzed according to coronary status (one‐, two‐, and three‐VD).

**Results:**

A total of 1886 patients (mean age 62.2 ± 14.0 year; 74% of male) were included. Patients with MVD (two or three‐VD) represented 53.7%. They were older with higher cardiovascular risk factor profile. At 1 year, the rate of major adverse cardiovascular events (MACE, defined as all‐cause death, stroke or re‐MI) was 10%, 12%, and 12% in one‐, two, and three‐VD respectively (*p* = .28). In multivariable adjusted Cox proportional hazard regression model, two‐ and three‐VD were not associated with higher rate of MACE compared to patients with single VD (HR, 1.09; 95%CI 0.76–1.56 for two‐VD; HR, 0.74; 95%CI 0.48–1.14 for three‐VD).

**Conclusions:**

MVD still represents an important proportion of STEMI patients but their prognoses were not associated with worse clinical outcomes at 1‐year compared with one‐VD patients in a modern reperfusion area and secondary medication prevention.

## 1 INTRODUCTION

ST‐elevation myocardial infarction (STEMI) with multivessel disease (MVD) represents between 40% and 65% of cases.^1^, ^2^, ^3^, ^4^ The primary objective of percutaneous coronary intervention (PCI) in these patients is to restore epicardial flow in the culprit vessel and normalize myocardial perfusion.^1^ Revascularization of non‐culprit lesion is still debated. However, the pathophysiological process of coronary artery disease (CAD) in myocardial infarction (MI) is not limited to the culprit vessel and MVD in STEMI patients is usually associated with worse clinical outcome including higher mortality compared with patients with single‐VD.^1^, ^2^, ^3^, ^4^

Several sources, including registries specific to acute myocardial infarction (AMI) and large administrative or billing databases, have shown a decrease in mortality in patients with STEMI over the past 30 years.^5^, ^6^, ^7^, ^8^, ^9^, ^10^, ^11^, ^12^, ^13^, ^14^ This decline is attributed to several factors (i.e., increased use and improved delivery of reperfusion therapy, in particular primary PCI, temporal changes in patient population characteristics over the period, increased use and improved delivery of recommended secondary prevention …).^5^, ^6^, ^7^, ^8^, ^9^, ^10^, ^11^, ^12^, ^13^, ^14^ To our knowledge, the impact of MVD on clinical outcomes in STEMI patients has not been assessed specifically after these changes.

The aim of our study is to assess the impact of MVD on major adverse cardiovascular events (MACE) at 1‐year in a modern reperfusion area and secondary medication prevention using the CRAC (Club Régional des Angioplasticiens de la région Centre) France PCI registry.

## 2 METHODS

### 2.1 Study population

The CRAC registry, created in 2014, brings together the six interventional cardiology centers of the Centre Val de Loire region and integrated the Clermont Ferrand University Hospital since 2016 to become the CRAC‐France PCI registry. It is an observational prospective multicenter registry, which includes all patients undergoing coronary angiography or coronary angioplasty in each participant center. The methods used for this registry have been detailed previously.^15^, ^16^

For the present analysis, we enrolled all consecutive STEMI patients undergoing PCI within 24 h of symptoms onset between January 1, 2014 and June 30, 2016 in the six ICCs which had been part of the CRAC registry (n = 1886). One ICC was excluded because of incomplete data. Non‐culprit lesion was defined as ≥50% diameter stenosis by visual estimate in at least one non‐infarct related vessel. Patient characteristics, management, and outcomes were analyzed according to coronary status (i.e. one‐, two‐, vs. three‐VD). To define CAD extent, all three coronary arteries were assigned one point each and two points for left main coronary artery (LMCA) whatever the status of left anterior descending and left circumflex, resulting in a maximum score of 3 (i.e., 3‐VD) in patients without a history of coronary artery bypass grafting (CABG). Patients with previous CABG were considered as three‐VD (n = 25). Multivessel CAD was defined as 2‐ or 3‐VD. Complete myocardial revascularization was considered in our analyses if the additional procedure was performed before discharge or during the first 3 months after index event. Complete myocardial revascularization was defined by successful PCI of all non‐culprit lesion(s) (i.e., restoration of blood supply to the myocardium). The primary endpoint of the study was a composite of MACE at 1‐year defined as all‐cause death, re‐MI, or stroke.

### 2.2 Data collection

The anonymous database includes up to 150 variables per procedure with hospital follow‐up data and at 1 year for any coronary angioplasty and pre‐hospital data for STEMI <24 h.^15^, ^16^ It includes demographic characteristics (age, sex, body mass index), risk factors (hypertension, diabetes mellitus, smoking, high cholesterol, family history of coronary heart disease, obesity), medical history and clinical presentation. Data on pre‐admission pathways are collected by emergency physicians: calls to emergency medical services (EMS; numbers 15, 18, or 112), physical location of the patient at the time of pain onset, timing of pain onset, CME (defined as first qualified ECG) and revascularization, type and number of medical contacts and first hospital admission. Procedural data are progressively completed by the cath lab staff (nurse, radiology manipulator, and interventional cardiologists) throughout the examination, from the patient's admission to discharge. The data are anonymized before being automatically transferred to the central database of CRAC‐France PCI.

Patient follow‐up was conducted by local research technicians on site at the participating centers. Major complications such as death, intrastent thrombosis, re‐MI, unplanned coronary revisions, major hemorrhage (BARC 3), and stroke were collected at discharge and after 1 year (telephone contact), in the form of antithrombotic therapy and cardiac rehabilitation. The 1‐year telephone call was made with direct access to medical and administrative information, which simplified and accelerated the process. In addition, the data collected was limited, focusing on MACE complications (which were expected to be low). Follow‐up at 1‐year was obtained in 95%. The data are anonymized before being automatically transferred to the central database of CRAC‐France PCI.

### 2.3 Ethical consideration

The study was conducted according to contemporary clinical practice guidelines and French regulations (Advisory Committee on Information Processing in Material Research in the Field of Health no. 13.245). The French Persons Protection Committee (IRB00003888) approved the study protocol (no. 15–231). Data file collection and storage were approved by the French National Commission for Data Protection and Liberties (no. 2014–073). All patients were informed of the aims of the survey. All included patients gave their informed consent to participate before data collection.

### 2.4 Statistical analysis

Continuous variables are reported as means (*SD*s) or medians and interquartile ranges (IQR), when appropriate. Discrete variables are described as counts and percentages. Groups were compared by analysis of variance for continuous variables and *χ* ^2^ (or Fisher exact tests) for discrete variables. Hazard ratios (HR) are presented with their 95% confidence intervals (CI). Survival curves were estimated using the Kaplan Meier estimators and compared using log rank tests. The rates of MACE at 1‐year were analyzed according to number of VD, and the impact of MVD (i.e., two‐ or three‐VD) was compared using a multivariate backward stepwise Cox analysis with a threshold of 0.10 for variable elimination, among the different risk groups. Variables included in the final models were selected ad hoc, based on their physiological relevance and potential to be associated with outcomes; they comprised age, gender, risk factors, comorbidities, year, and management. Two sensibility analyzes were performed in survival population after index hospitalization; and, in MVD patients according to complete myocardial revascularization. In addition, we repeated the multivariate analysis using a composite endpoint focused on cardiac clinical outcomes (i.e., cardiac death, MI, in stent thrombosis and urgent myocardial revascularization) to better evaluate the role of MVD on specific cardiac clinical outcomes. Analyses were repeated using forward stepwise analysis to check the consistency of the results. Statistical analyses were performed using IBM SPSS 26.0 (IBM SPSS Inc). For all analyses, 2‐sided *p* values <.05 were considered significant.

## 3 RESULTS

### 3.1 Patient characteristics

Figure S1 shows a flow chart for patient recruitment. Briefly, out of 11 883 patients undergoing PCI included in the CRAC‐France PCI registry over the period, 1886 STEMI ≤24 h patients treated by PCI with available medical information were selected for the present analysis. The mean age of the population was 62.6 ± 14.0 years (74% of male). MVD represents 53.7% of patients. Patient characteristics are presented in Table 1 according to coronary status (i.e. one‐, two‐, or three‐VD). Overall, cardiovascular risk‐profile progressively increased from patients with one‐VD to three‐VD. Patients with MVD were older with more risk‐factors (except for smoking status) and co‐morbidities. They had more previous MI and myocardial revascularization.

**TABLE 1:** Baselines characteristics

	All patients (n = 1886)	1‐VD (n = 873)	2‐VD (n = 623)	3‐VD (n = 390)	*p* value
Age (years)	62.62 ± 14.0	60.0 ± 14.3	63.0 ± 13.3	67.3 ± 13.6	<.001
Male	1115 (74)	524 (73)	371 (77.5)	220 (73)	.16
BMI (Kg/m^2^)	26.7 ± 4.4	26.5 ± 4.4	27.1 ± 4.4	26.5 ± 4.3	.06
Risk factors					
Hypertension	568 (38)	222 (31)	205 (43)	141 (47)	<.001
Diabetes	183 (12)	62 (9)	79 (16.5)	42 (14)	<.001
Hypercholesterolemia	574 (38)	241 (33)	199 (41.5)	134 (44.5)	.001
Current smoking	586 (39)	327 (45)	172 (36)	87 (29)	<.001
Family history	310 (21)	139 (19)	103 (21.5)	68 (23)	.42
Medical history					
Prior MI	96 (6)	38 (5)	32 (7)	26 (9)	.02
Prior PCI	158 (10.5)	59 (8)	61 (13)	38 (13)	.02
Prior CABG	25 (1.3)	—	—	25 (6)	—
History of stroke	37 (2.5)	13 (2)	17 (3.5)	7 (2)	.37
Peripheral artery disease	50 (3)	22 (3)	14 (3)	14 (5)	.42
Chronic renal failure	23 (1.5)	6 (0.8)	8 (2)	9 (3)	.06

*Note:* Values are expressed as mean (±*SD*) or number (percentage).

Abbreviations: BMI, body mass index; CABG, coronary artery bypass grafting; MI, myocardial infarction; PCI, percutaneous coronary intervention.

### 3.2 In‐hospital management and duration of dual antiplatelet therapy

All patients had an invasive strategy and were referred to a cardiac catheterization laboratory. Coronary angiogram showed that the site of the culprit lesion differed according to coronary status (mainly in the left anterior descending artery for patients with one‐VD; and, mainly in the right coronary artery for patients with MVD) (Table 2). Patients with MVD had more diffuse CAD including longer lesions with smaller diameters. The Syntax score gradually increased between patients with one‐VD to three‐VD (one‐VD: 8.9 ± 5.9; two‐VD: 13.0 ± 7.9; three‐VD: 19.0 ± 10.7, *p* < .001). The rates of TIMI score 0/1 of the culprit lesion before primary PCI was similar in all groups. Procedural characteristics are detailed in Table 2. No difference was observed related to vascular approach and the size of the sheath according to all groups. Primary PCI was performed in 98% of the population. Thrombus aspiration was mainly used in patient with one‐VD. Drug‐eluting stents were used similarly in all groups, but the number of stents implanted was higher in patients with MVD. Proportion of PCI success was similar in all groups (93% in overall population) as was the rate of TIMI score 2/3 post‐PCI (95% in overall population). Complete myocardial revascularization was performed preferentially before discharge in 26% and 30% of patients with two‐ and three‐VD respectively. Finally, the quantity of contrast and radiation exposure was higher in MVD patients.

**TABLE 2:** Baselines angiographic, echocardiographic, and procedural characteristics

	All patients (n = 1886)	1‐VD (n = 873)	2‐VD (n = 623)	3‐VD (n = 390)	*p* value
Angiographic characteristics					
Approach					.69
Femoral	175 (9)	78 (9)	56 (9)	41 (10.5)	
Radial	1707 (90.5)	794 (91)	565 (91)	348 (89)	
Humeral	2 (0.2)	1 (0.1)	2 (0.4)	0 (0)	
Sheath					.41
5 French	19 (1)	12 (1)	3 (0.5)	4 (1)	
6 French	1833 (97)	843 (97)	608 (98)	382 (98)	
7 French	18 (1)	9 (1)	7 (1)	2 (0.5)	
Culprit lesion					<.001
Left main	14 (0.7)	0 (0)	9 (1)	5 (1)	
LAD	758 (40)	407 (47)	230 (37)	121 (31)	
LCX	277 (15)	113 (13)	105 (17)	59 (15)	
RCA	796 (42)	335 (38)	271 (42.5)	196 (49)	
CABG	5 (0.3)	0 (0)	0 (0)	5 (1)	
Stenosis (culprit lesion)					.26
100%	1068 (57)	516 (59)	349 (56)	203 (52)	
90%–99%	419 (22)	187 (21)	135 (22)	97 (25)	
70%–90%	305 (16)	128 (15)	109 (17.5)	68 (17)	
50%–70%	60 (3)	25 (3)	23 (4)	12 (3)	
Length (culprit lesion)					.02
<10 mm	228 (12)	109 (12.5)	82 (13)	38 (10)	
10–20 mm	1120 (59)	542 (62)	353 (57)	225 (58)	
>20 mm	493 (26)	197 (23)	180 (29)	116 (30)	
Diameter (culprit lesion)					.009
<2.5 mm	62 (3)	24 (3)	14 (2)	24 (6)	
2.5–3.0 mm	1061 (56)	476 (54)	359 (58)	226 (58)	
>3.0 mm	730 (39)	357 (41)	243 (39)	130 (33)	
Restenosis	93 (5)	32 (4)	41 (6.5)	20 (5)	.25
TIMI pre‐PCI					.28
0/1	1072 (57)	516 (59)	354 (57)	202 (52)	
2/3	781 (42)	341 (39)	262 (42)	178 (46)	
SYNTAX score	12.3 ± 8.7 N = 1884	8.9 ± 5.9 N = 872	13.0 ± 7.9 N = 622	19.0 ± 10.7 N = 390	<.001
Echocardiographic data					
LVEF	53.9 ± 12.8	54.7 ± 12.2	54.6 ± 12.6	48.3 ± 16.2	.34
Type of revascularization					
PCI alone	1853 (98)	858 (98)	619 (99)	376 (96)	<.001
CABG alone	3 (0.1)	1 (0.1)	0 (0)	2 (0.5)	
PCI and CABG	10 (0.5)	0 (0)	1 (0.2)	9 (2)	
Medical therapy alone	14 (0.7)	9 (1)	2 (0.3)	3 (0.8)	
Procedural characteristics (culprit lesion)					
Thromboaspiration	835 (45)	435 (51)	270 (44)	130 (34)	<.001
BMS	307 (17)	147 (17)	109 (18)	51 (13.5)	.18
DES	1266 (68)	574 (67)	421 (68.5)	271 (71.5)	.30
Balloon alone	154 (8)	69 (8)	45 (7)	40 (11)	.19
Number of stents implanted	1.22 ± 0.80	1.11 ± 0.70	1.29 ± 0.83	1.35 ± 0.94	.02
Length of stent(s)	20.3 ± 9.0	20.0 ± 8.5	20.6 ± 9.5	20.7 ± 9.1	.32
TIMI pre‐PCI					.83
0/1	56 (3)	23 (3)	19 (3)	14 (4)	
2/3	1787 (95)	830 (95)	592 (95)	365 (94)	
PCI success	1755 (93)	817 (94)	581 (93)	357 (91.5)	.28
Complete myocardial revascularization					<.001
Before discharge	1039 (55)	830 (95)	118 (19)	91 (23)	
After discharge	93 (5)	26 (3.0)	43 (7)	24 (6)	
Circulatory support	59 (3)	25 (3)	20 (3)	14 (4)	.70
Contrast (ml)	147 ± 61	139 ± 56	149 ± 60	157 ± 72	<.001
Scopie (min)	8.8 ± 6.8	8.1 ± 6.4	9.1 ± 7.1	10.0 ± 7.3	<.001
PDS (cGyxm^2^)	6777 ± 91 065	4323 ± 7355	4927 ± 4589	15 217 ± 199 796	.12

*Note:* Values are expressed as mean (±*SD*) or number (percentage).

Abbreviations: BMI, body mass index; BMS, bare metal stent; CABG, coronary artery bypass grafting; DES, drug eluting stent; LAD, left anterior descending artery; LCX, left circumflex coronary artery; MI, myocardial infarction; PDS, produit dose x surface; PCI, percutaneous coronary intervention; RCA, right coronary artery.

Antithrombotic treatment used before admission and medications prescription at discharge are given in Table S1. The choice of antithrombotic treatment (i.e., antiplatelet and anticoagulant) did not differ according to coronary status. Ticagrelor was the P2Y12 inhibitor mostly prescribed whatever the coronary status (70%). At discharge, proportions of recommended secondary prevention medications (i.e., angiotensin converting enzyme inhibitors [ACE‐I] or angiotensin receptor blocker [ARB], statins and betablockers) progressively increased from one‐VD to three‐VD.

Finally, duration of dual antiplatelet therapy after AMI was mainly ≥12 months whatever the coronary status (Figure S2).

### 3.3 In‐hospital complications and clinical outcomes at 1 year

In‐hospital complications (i.e., re‐MI, stroke, in‐stent thrombosis, and major bleeding) did not differ according to coronary status as the case for the rate of in‐hospital death (6% in the overall population) (Table 3).

**TABLE 3:** In‐hospital complications, clinical outcomes, and antithrombotic used at 1‐year

	All patients (n = 1886)	1‐VD (n = 873)	2‐VD (n = 623)	3‐VD (n = 390)	*p* value
In‐hospital complications					
Death	108 (6)	44 (5)	42 (7)	22 (6)	.06
Myocardial infarction	20 (1)	6 (0.7)	9 (1)	5 (1)	.30
Stroke	6 (0.4)	2 (0.2)	1 (0.2)	3 (0.8)	.12
In‐stent thrombosis	22 (1)	8 (0.9)	9 (1)	5 (1)	.75
Major bleeding (BARC ≥3)	40 (2)	12 (1)	17 (3)	11 (3)	.19
Clinical outcomes at 1‐year					
MACE	196 (11)	79 (10)	70 (12)	47 (12)	.28
Death	169 (9)	64 (7)	64 (10)	41 (10.5)	.006
Myocardial infarction	27 (1.5)	13 (1.5)	10 (2)	4 (1)	.13
Stroke	8 (0.5)	4 (0.5)	0 (0)	4 (1)	.01
In‐stent thrombosis	14 (0.8)	9 (1)	1 (0.2)	4 (1)	.01
Major bleeding (BARC ≥3)	28 (2)	15 (2)	7 (2)	6 (2)	.10
Urgent myocardial revascularisation^a^	83 (4)	25 (3)	24 (5)	29 (7.5)	<.001
Antithrombotic at 1‐year					
Aspirin	1816 (96)	841 (96)	598 (96)	377 (97)	.04
Clopidogrel	234 (12)	95 (11)	82 (13)	57 (15)	.02
Prasugrel	70 (4)	32 (4)	22 (3.5)	16 (4)	.08
Ticagrelor	583 (31)	259 (30)	203 (33)	121 (31)	.08
Oral anticoagulant	267 (14)	112 (13)	92 (15)	63 (16)	.13

*Note:* Values are expressed as number (percentage).

Abbreviation: BARC, Bleeding Academic Research Consortium.

^a^ Percutaneous coronary intervention or coronary artery bypass graft.

**FIGURE 1:**
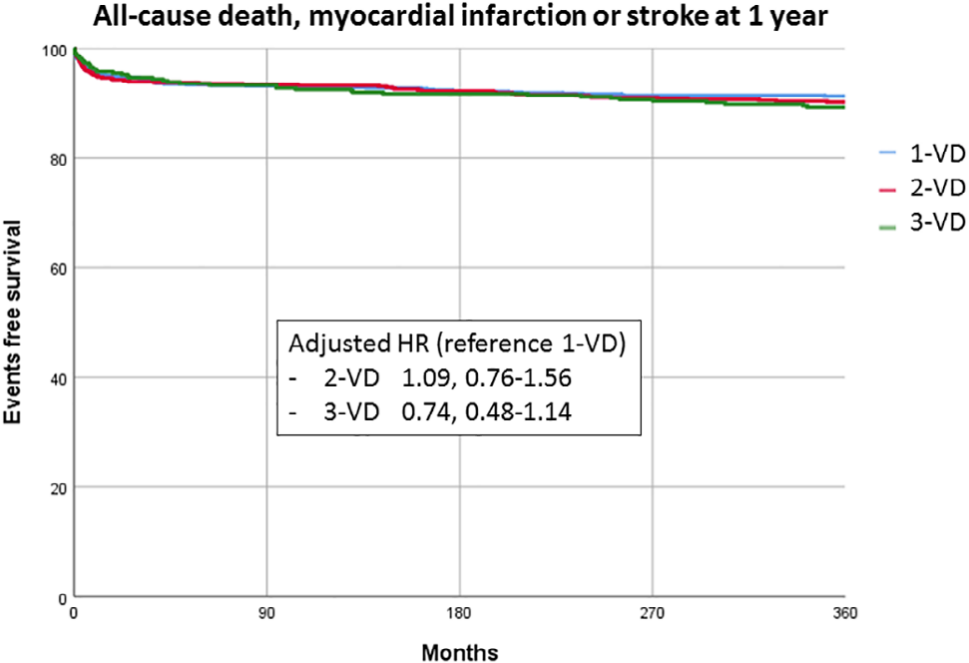
One‐year events‐free survival according to coronary status

The proportion of MACE at 1‐year was 11% in the overall population and was not significantly different in patients with one‐VD (10%), two‐VD (12%), and three‐VD (12%) (*p* = .28) (Figure 1). The proportion of cardiac death, MI, in stent thrombosis and urgent myocardial revascularization was however higher in patients with MVD compared with patients with single‐VD (three‐VD: 16.8%, two‐VD: 15.3%, one‐VD: 10.6%; *p* = .004). The rate of death was higher in patients with MVD compared with one‐VD patients (10% vs. 7%), while the rate of stroke and re‐MI were similar in all groups. Major bleeding was observed similarly in all groups (2%). Finally, the use of urgent myocardial revascularization was higher in MVD patients (one‐VD: 3%; two‐VD: 5%; three‐VD: 7.5%, *p* < .001).

In patients with MVD, the rate of MACE at 1 year was 5.2% in patients with complete myocardial revascularization and 14.4% in those without complete myocardial revascularization (*p* < .001).

The multivariable adjusted Cox proportional hazard regression model showed that two‐ and three‐VD were not associated with worse prognosis at 1‐year compared to patients with single VD (HR, 1.09; 95%CI 0.76–1.56 for two‐VD; HR, 0.74; 95%CI 0.48–1.14 for three‐VD) (Table 4). In this model, age, current smoking, previous CABG, chronic kidney disease, diameter of culprit lesion >3.0 mm and anterior MI were associated with higher rates of MACE, while the use of new P2Y12 inhibitors, statins, TIMI score 2/3 post PCI, and LVEF >40% at discharge were protectors. Similar results were found regarding cardiac death, MI, in stent thrombosis and urgent myocardial revascularization at 1‐year (HR, 1.03; 95%CI 0.58–1.83 for two‐VD; HR, 0.76; 95%CI 0.42–1.39 for three‐VD). Complete myocardial revascularization was not associated with better clinical outcome (HR, 1.00; 95%CI 0.54–1.86). Similar results were found after excluding in hospital death (data not shown). Finally, in MVD patients, complete myocardial revascularization was not associated with lower MACE (HR, 1.09; 95%CI 0.58–2.08).

**TABLE 4:** Major adverse cardiovascular events at 1‐year in multivariate analysis

Variables	Hazard ratio 95%, confidence interval	*p*‐value
Age, per year	1.03 (1.02–1.05)	<.001
Previous CABG	3.08 (1.12–8.48)	.03
Chronic kidney disease	2.15 (1.07–4.32)	.03
Current smoking	1.72 (1.05–2.82)	.03
Anterior MI	1.41 (1.02–1.96)	.04
Diabetes	0.98 (0.63–1.54)	.94
Dyslipidemia	0.78 (0.55–1.09)	.15
Hypertension	0.92 (0.63–1.33)	.66
Previous stroke	1.09 (0.52–2.30)	.82
Body mass index >30	1.15 (0.74–1.79)	.54
Current smoking	1.72 (1.05–2.82)	.03
Previous MI	1.08 (0.56–2.06)	.83
Sex (male)	1.05 (0.71–1.56)	.81
Peripheral artery disease	0.65 (0.32–1.33)	.24
Antiplatelet therapy (reference: Prasugrel or Ticagrelor)		
Clopidogrel	2.57 (1.55–4.27)	<.001
Statins	0.40 (0.16–0.97)	.04
Betablockers	0.63 (0.27–1.44)	.27
ACE‐I or ARB	1.04 (0.48–2.27)	.92
GPIIBIIIA inhibitors	0.92 (0.64–1.33)	.66
Anticoagulant (reference: LMWH)		
UFH	0.87 (0.60–1.26)	.47
Bivalirudin	5.08 (1.52–16.97)	.008
Fondaparinux	3.69 (0.50–27.0)	.20
TIMI post PCI (3/2 vs. 1/0)	0.56 (0.33–0.95)	.03
Diameter of culprit lesion ≥ 3.0 mm	1.54 (1.01–2.35)	.04
Length of culprit lesion (reference: <20 mm)		
≥20 and <30 mm	1.27 (0.71–2.28)	.43
≥30 mm	1.35 (0.73–2.48)	.34
Thromboaspiration	0.84 (0.60–1.18)	.32
Angiography results (reference: 1‐VD)		
2‐VD	1.09 (0.76–1.56)	.66
3‐VD	0.74 (0.48–1.14)	.17
Complete myocardial revascularization	1.00 (0.54–1.86)	.99
LVEF >40% at discharge	0.33 (0.15–0.70)	.004

Abbreviations: CABG, coronary artery bypass grafting; LMWH, low molecule weight heparin; LVEF, left ventricular ejection fraction; MI, myocardial infarction; PCI, percutaneous coronary intervention; UFH, unfractioned heparin; VD, vessel disease.

## 4 DISCUSSION

The main findings of this study are that STEMI with MVD currently represents approximately 50% of patients admitted to a cardiac catheterization laboratory. Patients with MVD received more aggressive secondary medication prevention. Finally, the presence of two or three‐VD associated with the culprit lesion was not associated with higher rate of MACE (or a composite endpoint combining cardiac death, MI, in stent thrombosis and urgent myocardial revascularization) at 1 year compared with those with culprit lesion alone (i.e., one‐VD).

### 4.1 Improvement of survival among STEMI patients

During the last 30 years, several registries specific to AMI and large administrative or billing databases have shown a decrease in mortality in patients with STEMI.^5^, ^6^, ^7^, ^8^, ^9^, ^10^, ^11^, ^12^, ^13^, ^14^ Most benefits in short‐ and long‐term outcomes in patients with STEMI were related to the uptake and increased use of new and, by time, established interventional and medical treatments.^1^ The improvement in hospital survival was mainly related to the increased use of reperfusion treatment including primary PCI. Concerning the 1‐year outcomes, the results indicated that not only reperfusion and revascularization but also the broad uptake and prescription of aspirin, P2Y12‐inhibition, beta‐blockade, ACE/A2 inhibition, and statins contributed to the lower rates of events.^1^ Using the FAST‐MI registries, Danchin et al have demonstrated that the reduction of mortality parallels improvements in care and was also associated with a substantial change in the patient risk profile.^14^

Improved survival among STEMI patients was observed in all categories of patients over the last 30 years.^5^, ^6^, ^7^, ^8^, ^9^, ^10^, ^11^, ^12^, ^13^, ^14^ To our knowledge, there is no recent comparison focused on patients with MVD compared with those with single‐VD. Using clinical trials data from STEMI patients with myocardial revascularization and MVD from 2008, it is possible to estimate the rate of MACE in this population and the trends over the last 10‐year period.^17^, ^18^, ^19^, ^20^, ^21^ In the Preventive Angioplasty in Acute Myocardial Infarction (PRAMI) trial the rate of MACE (death from cardiac causes, non‐fatal MI, or refractory angina; mean follow‐up of 23 months) was 9.0% in preventive PCI and 22.9% in no preventive PCI.^17^ Comparisons of clinical outcomes according to trials are difficult because of different primary outcomes and follow‐up duration. However, the proportion of MACE seems to decrease from 2008 on. In the most recent study (Complete vs. Culprit‐Only Revascularization Strategies to Treat Multivessel Disease after Early PCI for STEMI [COMPLETE] trial), the rate of MACE (Cardiovascular death, MI, or ischemia‐driven revascularization; median follow‐up of 3 years) was 3.1% in complete revascularization strategy to 6.2% in culprit‐lesion‐only PCI.^21^ These data suggest that the prognosis of MVD in STEMI patients has changed over the period and, now it is probably close to that of patients with single‐VD. In our main analysis, the rate of MACE at 1‐year did not differ according to coronary status after adjustment.

### 4.2 Management of STEMI with MVD

Primary PCI is the preferred reperfusion strategy in patients with STEMI within 12 h of symptom onset.^22^ MVD is commonly reported (in approximately 50%) in this population as observed in our study (53.7%). A series of successful clinical trials have proven the improved survival and lower morbidity with complete myocardial revascularization compared to culprit‐lesion‐only PCI in STEMI patients with MVD.^17^, ^18^, ^19^, ^20^, ^21^ This has led to very consistent global treatment recommendations. Therefore policies of complete myocardial revascularization have increased over the last 10 years even the timing is conflicting.^14^, ^22^

In addition, our data show that patients with MVD received more aggressive secondary medication prevention at discharge and the proportion of DAPT at 1 year was numerically higher in this population.^1^, ^22^ This represents certainly an important point to explain our results. Finally, the use of new generation drug‐eluting stents associated with new P2Y12 inhibitors can reduce complications of PCI and improve the prognosis of these patients.^22^

### 4.3 Limitations

As in any observational study, there are limitations to our analysis. Only STEMI ≤24H patients admitted to a cardiac catheterization laboratory were included, which represents a selection bias. Several data are missing in the database to better define the study groups such as atrial fibrillation (AF). Recent data have shown that patients with AF have generally less severe CAD compared to non‐AF ones.^23^ In addition, the use of secondary medication prevention are only available during the first 24 h. Finally, the clinical follow‐up duration is limited, and we cannot exclude that the prognosis will be similar in all groups after 1 year.

## 5 CONCLUSIONS

MVD in STEMI patients still represents half of the patients despite a substantial change in the patient risk profile. However, the prognosis of patients with two or three VD is not associated with higher rate of MACE (or cardiac clinical outcomes) at 1‐year compared to patients with single‐VD.

## CONFLICT OF INTEREST

None of the companies had a role in the design and conduct of the study, data collection, and management. Etienne Puymirat has received research grants/consultant fees/lectures fees: Amgen, Astra‐Zeneca, Abbott, Bayer, Biotronik, BMS, Boehringer Ingelheim, Daiichi‐Sankyo, Lilly, MSD, Novartis, Sanofi; Nicolas Danchin has received grants, personal fees, and non‐financial support from Amgen, AstraZeneca, Bayer, Bristol‐Myers Squibb, and Sanofi and personal fees from Boehringer Ingelheim, Intercept, Merck Sharp & Dohme, Novo Nordisk, Pfizer, and UCB Pharmaceuticals. Guillaume Cayla has received research grants/consultant fees/lectures fees from Amgen, AstraZeneca, Abbott, Bayer, Biotronik, Bristol‐Myers Squibb, Pfizer, Sanofi‐Aventis; Gilles Montalescot reports consulting or speaker fees from Abbott, AIM group, Amgen, Actelion, American College of Cardiology Foundation, Astrazeneca, Axis‐Santé, Bayer, Boston‐Scientific, Bristol‐Myers Squibb, Beth Israel Deaconess Medical, Brigham Women's Hospital, Fréquence Médicale, ICOM, Idorsia, Elsevier, Fédération Française de Cardiologie, Fréquence Médicale, ICAN, Lead‐Up, Menarini, Medtronic, MSD, Novo‐Nordisk, Pfizer, Quantum Genomics, Sanofi‐Aventis, SCOR global life, Servier, WebMD; Ariel Nakache, Christophe Saint Etienne, Pierre Marcollet, Olivier Fichaux, Marie‐Pascale Decomis, Stephan Chassaing, Philippe Commeau, Hakim Benamer, Rene Koning, Pascal Motreff, and Grégoire Rangé have no conflict of interest.

## Supporting information


**Table S1**. Antithrombotic used during prehospital management and medications prescribed at discharge.
**Figure S1**: Flow chart.
**Figure S2**: Dual antiplatelet therapy duration according to number of vessel disease.

## Data Availability

Data available on request from the authors. The data that support the findings of this study are available from the corresponding author upon reasonable request.
