# Supplementary material for: Is coronary multivessel disease in acute myocardial infarction patients still associated with worse clinical outcomes at 1‐year?
Source: Clin Cardiol. 2021 Feb 14;44(3):429–37. doi: 10.1002/clc.23567 (PMC7943894; doi:10.1002/clc.23567)

**Supplemental Material:**

**eTable 1. Antithrombotic used during prehospital management and medications prescribed at discharge.**

**eFigure1. Flow chart.**

**eFigure 2. Dual antiplatelet therapy duration according to number of vessel disease.**

**eTable 1. Antithrombotic used during prehospital management and medications prescribed during the first 24 hours.**

|  | **All patients**  **(n=1886)** | **1-VD**  **(n=873)** | **2-VD**  **(n=623)** | **3-VD**  **(n=390)** | **P**  **value** |
| --- | --- | --- | --- | --- | --- |
| **Antithrombotic used during prehospital management** | | | | | |
| Aspirin | 1794 (95) | 824 (94) | 590 (95) | 380 (97) | 0.06 |
| Clopidogrel  Prasugrel  Ticagrelor | 308 (16)  111 (6)  1312 (70) | 130 (15)  57 (6.5)  610 (70) | 101 (16)  36 (6)  433 (69.5) | 77 (20)  18 (5)  269 (69) | 0.57 |
| UFH  LMWH  Bivalirudin  Fondaparinux | 642 (43)  663 (44)  20 (1)  7 (0.5) | 303 (42)  326 (45)  8 (1)  4 (0.6) | 204 (43)  216 (45)  5 (1)  1 (0.2) | 135 (45)  121 (40)  7 (2)  2 (0.7) | 0.79 |
| **Medications prescribed during the first 24 hours** | | | | | |
| Aspirin | 1874 (99) | 865 (99) | 621 (100) | 388 (100) | 0.40 |
| Clopidogrel  Prasugrel  Ticagrelor | 264 (14)  166 (9)  1324 (70) | 112 (13)  82 (9)  626 (72) | 82 (13)  53 (8.5)  440 (71) | 70 (18)  31 (8)  258 (66) | 0.10 |
| ACE-I or ARB | 882 (47) | 389 (45) | 297 (48) | 196 (50) | 0.02 |
| Statins | 1036 (55) | 454 (52) | 350 (56) | 232 (59.5) | 0.04 |
| Betablockers | 988 (52) | 435 (50) | 335 (54) | 218 (56) | 0.10 |

Values are expressed as number (percentage)

*ACE-I, angiotensin-converting enzyme inhibitor; ARB, angiotensin receptor blocker; LMWH, Low molecular weight heparin; UFH; unfractionated heparin*

**eFigure1. Flow chart.**

**
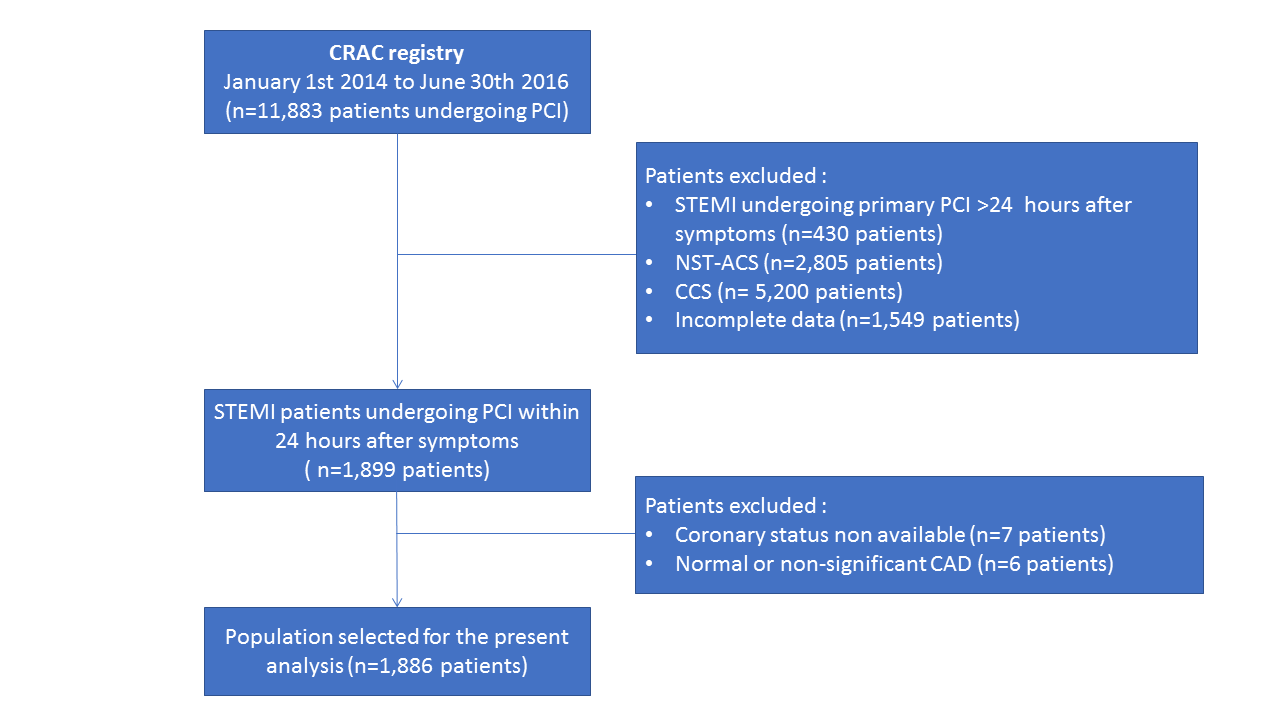
**

CAD, coronary artery disease; CCS, chronic coronary syndrome; PCI, percutaneous coronary intervention; NST-ACS, Non-ST-Acute coronary syndrome; STEMI, ST-elevation myocardial infarction

**eFigure 2. Dual antiplatelet therapy duration according to number of vessel disease.**

DAPT, dual antiplatelet therapy

VD, vessel disease


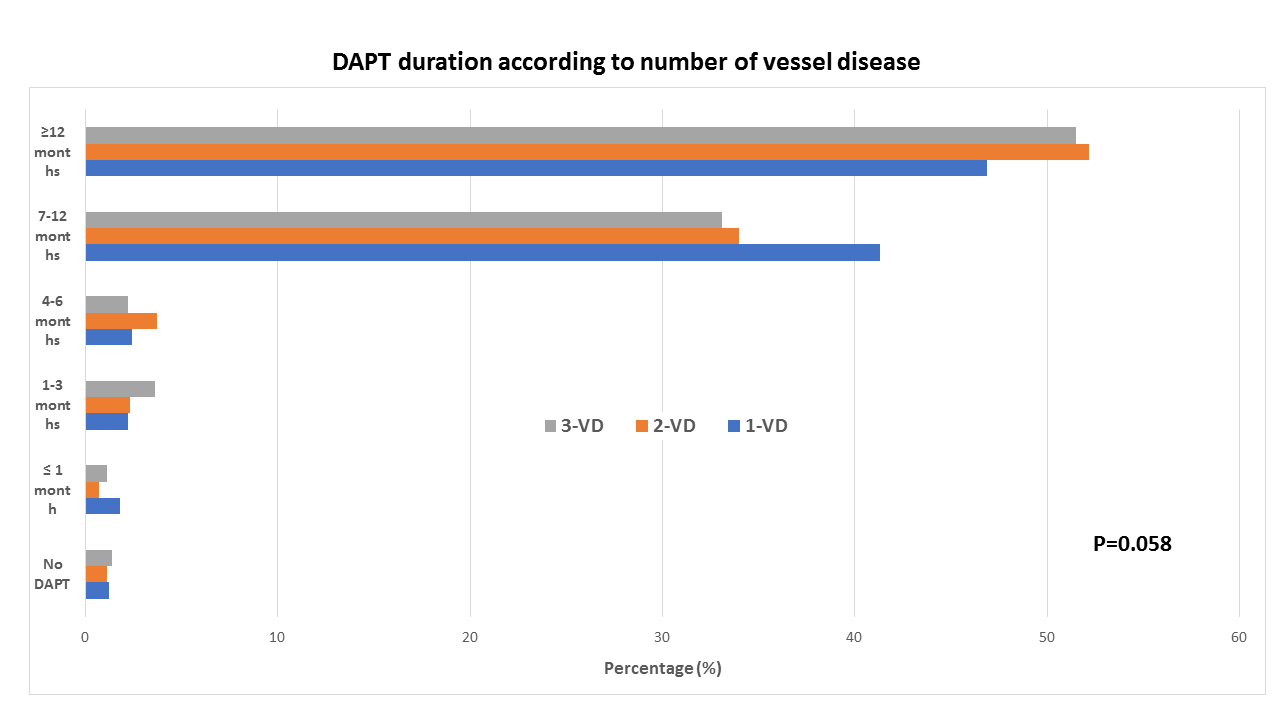

Supplement: Supplementary file 1 — Table S1. Antithrombotic used during prehospital management and medications prescribed at discharge. Figure S1: Flow chart. Figure S2: Dual antiplatelet therapy duration according to number of vessel disease. [file CLC-44-429-s001.docx]
